# Dr. George A. Bachmann

**Published:** 1918-02

**Authors:** 


**Dr. George A. Bachmann,** Rochester, died Nov. 21, age 49, from heart disease. He graduated from the New York Homeopathic Medical College in 1892.

